# Supplementary material for: High-resolution global recombination mapping in C. elegans reveals sexual dimorphisms shaped by meiotic chromosomal features and structures
Source: PLoS Genet. 2026 Jul 14;22(7):e1012237. doi: 10.1371/journal.pgen.1012237 (PMC13387615; doi:10.1371/journal.pgen.1012237)
Supplement: S1 Fig — (A) A histogram depicting the distribution of read coverage at marker sites across all individual wild-type samples. (B) A histogram depicting the distribution of read coverage at marker sites across all individual syp-2/ + samples. Vertical dashed line indicates the average across all samples. (PDF) [file pgen.1012237.s004.pdf]

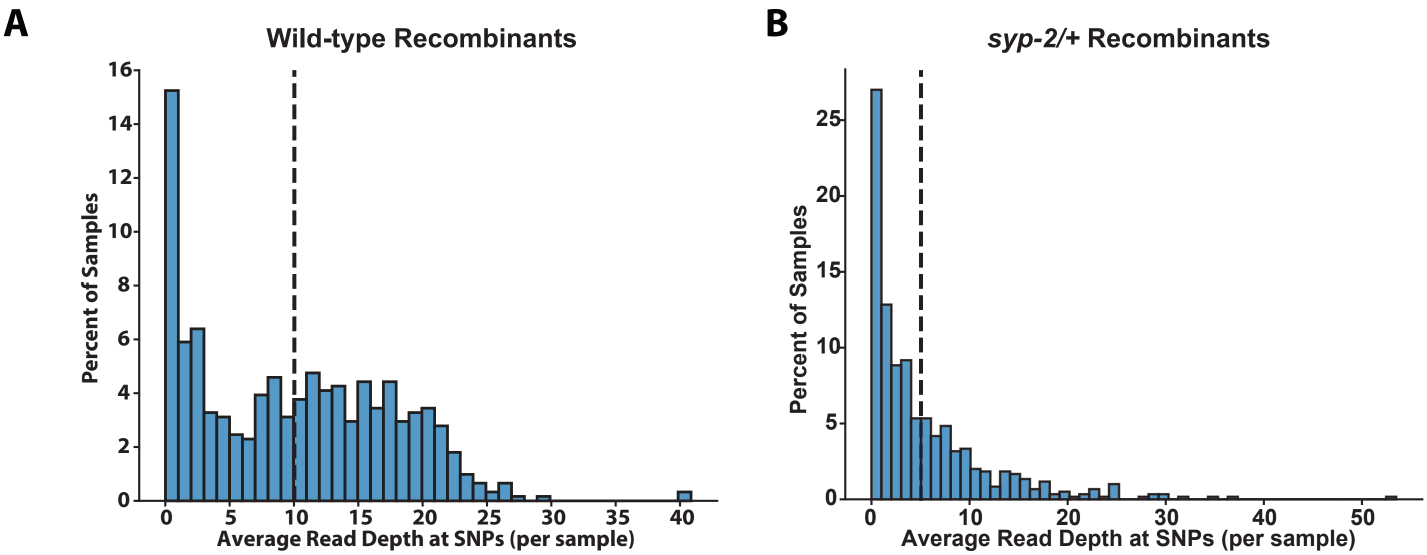

S1 Fig. Sequencing read depth of F2 recombinants. (A) A histogram depicting the distribution of read coverage at marker sites across all individual wild type samples. (B) A histogram depicting the distribution of read coverage at marker sites across all individual *syp-2*/+ samples. Vertical dashed line indicates the average across all samples.
