# Supplementary material for: High-resolution global recombination mapping in C. elegans reveals sexual dimorphisms shaped by meiotic chromosomal features and structures
Source: PLoS Genet. 2026 Jul 14;22(7):e1012237. doi: 10.1371/journal.pgen.1012237 (PMC13387615; doi:10.1371/journal.pgen.1012237)
Supplement: S2 Fig — (A) Data collection and preprocessing including Illumina whole-genome sequencing, read alignment, and SNP calling. (B) Schematic of Hidden Markov Model based reconstruction of recombinant chromosomes and subsequent crossover detection via supervised machine learning and random forest classification. (PDF) [file pgen.1012237.s005.pdf]

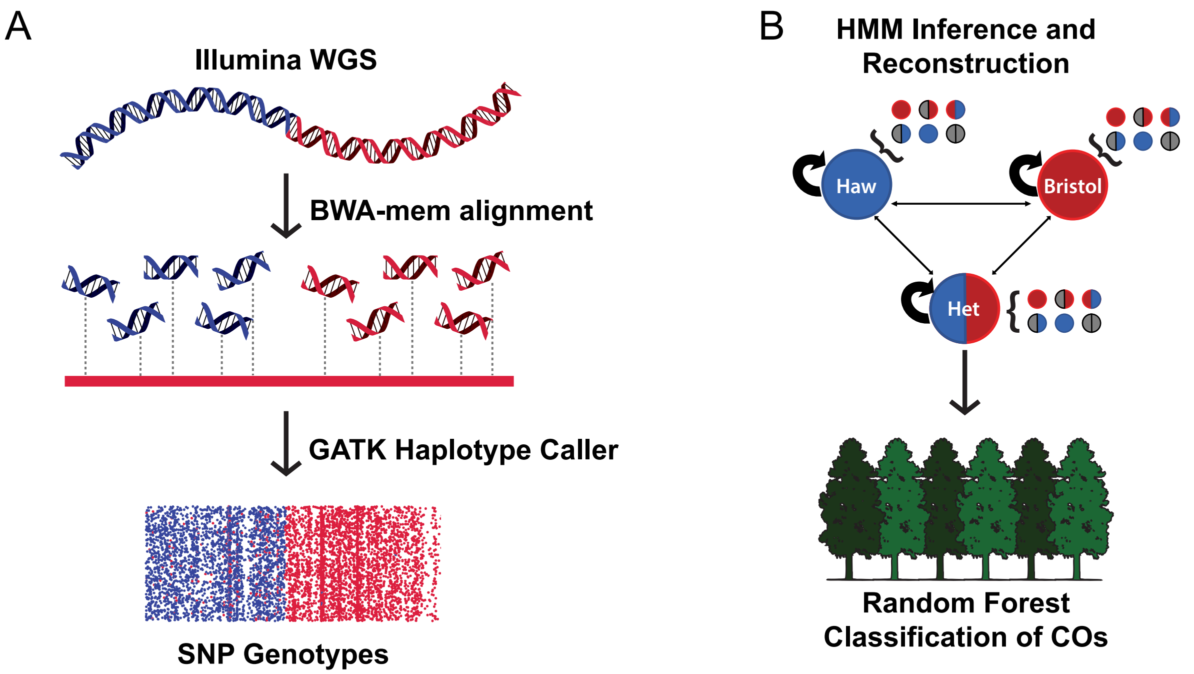

S2 Fig. Computational pipeline for processing of sequencing data. (A) Data collection and pre-processing including Illumina whole-genome sequencing, read alignment, and SNP calling. (B) Schematic of Hidden Markov Model based reconstruction of recombinant chromosomes and subsequent crossover detection via supervised machine learning and random forest classification.
