# Supplementary material for: High-resolution global recombination mapping in C. elegans reveals sexual dimorphisms shaped by meiotic chromosomal features and structures
Source: PLoS Genet. 2026 Jul 14;22(7):e1012237. doi: 10.1371/journal.pgen.1012237 (PMC13387615; doi:10.1371/journal.pgen.1012237)
Supplement: S3 Fig — (A) A bar chart indicating the number of noncrossovers (NCOs) identified in individual oocyte and spermatocyte genomes. (B) A bar chart showing the average length of NCO tracts in individual oocyte and spermatocyte genomes. Error bars indicated 95% confidence intervals. (PDF) [file pgen.1012237.s006.pdf]

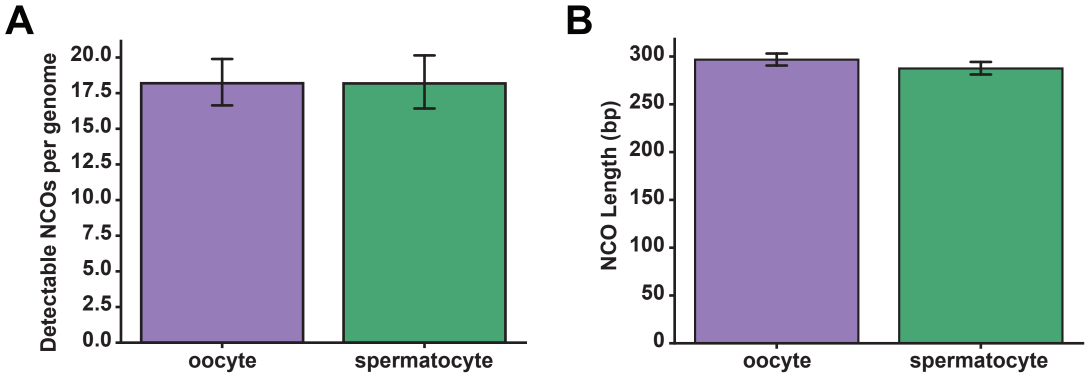

S3 Fig. Characteristics of detectable NCO events in wild-type germ cells. (A) A bar chart indicating the number of noncrossovers (NCOs) identified in individual oocyte and spermatocyte genomes. (B) A bar chart showing the average length of NCO tracts in individual oocyte and spermatocyte genomes. Error bars indicated 95% confidence intervals.
