# Supplementary material for: High-resolution global recombination mapping in C. elegans reveals sexual dimorphisms shaped by meiotic chromosomal features and structures
Source: PLoS Genet. 2026 Jul 14;22(7):e1012237. doi: 10.1371/journal.pgen.1012237 (PMC13387615; doi:10.1371/journal.pgen.1012237)
Supplement: S4 Fig — Scatterplot showing the correlation of oocyte and spermatocyte recombination rates in sliding windows of varying sizes. Kendall’s tau was calculated as the correlation coefficient, all p-values < 0.05 except 10 kb window size on chromosome V. Red line indicates the curve of best fit for each chromosome. (PDF) [file pgen.1012237.s007.pdf]

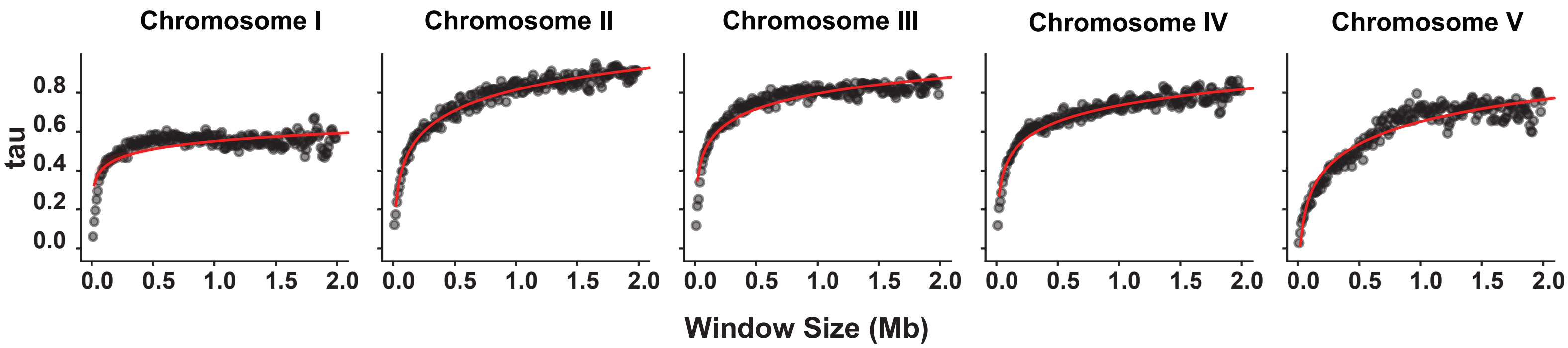

S4 Fig. Correlation of egg and sperm crossover rates at multiple scales. Scatterplot showing the correlation of oocyte and spermatocyte recombination rates in sliding windows of varying sizes. Kendall's tau was calculated as the correlation coefficient, all p-values < 0.05 except 10kb window size on chromosome V. Red line indicates the curve of best fit for each chromosome.
