# Supplementary material for: High-resolution global recombination mapping in C. elegans reveals sexual dimorphisms shaped by meiotic chromosomal features and structures
Source: PLoS Genet. 2026 Jul 14;22(7):e1012237. doi: 10.1371/journal.pgen.1012237 (PMC13387615; doi:10.1371/journal.pgen.1012237)
Supplement: S5 Fig — A bar chart showing the median distance from every detected crossover to the nearest cluster of DNA motifs bound by pairing center proteins. Asterisks indicate p < 0.05 by Kruskal-Wallis H-test followed by post-hoc Dunn’s test. Error bars indicate 95% confidence intervals. (PDF) [file pgen.1012237.s008.pdf]

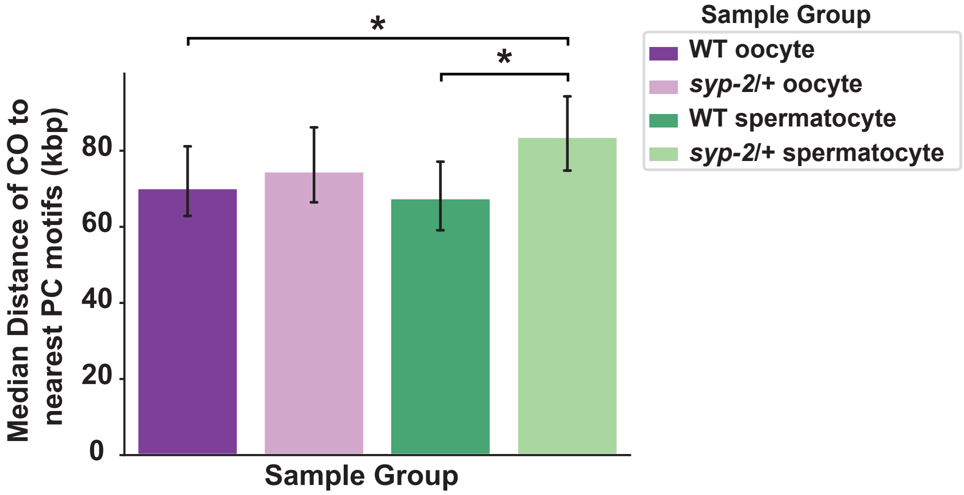

S5 Fig. Wild type and *syp-2*/+ crossovers associated with chromosomal pairing domains. A bar chart showing the median distance from every detected crossover to the nearest cluster of DNA motifs bound by pairing center proteins. Asterisks indicate $p < 0.05$ by Kruskal-Wallis H-test followed by post-hoc Dunn's test. Error bars indicate 95% confidence intervals.
