# Supplementary material for: High-resolution global recombination mapping in C. elegans reveals sexual dimorphisms shaped by meiotic chromosomal features and structures
Source: PLoS Genet. 2026 Jul 14;22(7):e1012237. doi: 10.1371/journal.pgen.1012237 (PMC13387615; doi:10.1371/journal.pgen.1012237)
Supplement: S6 Fig — (A) Heatmap showing the log2(fold) enrichment or depletion of oocyte crossovers with each sequence annotation. (B) Heatmap showing the log2(fold) enrichment or depletion of spermatocyte crossovers with each sequence annotation. All annotations were taken from the Ensembl ce11 genome annotation set and remapped to Libuda N2 Bristol genome assembly via LiftOff (See methods). (PDF) [file pgen.1012237.s009.pdf]

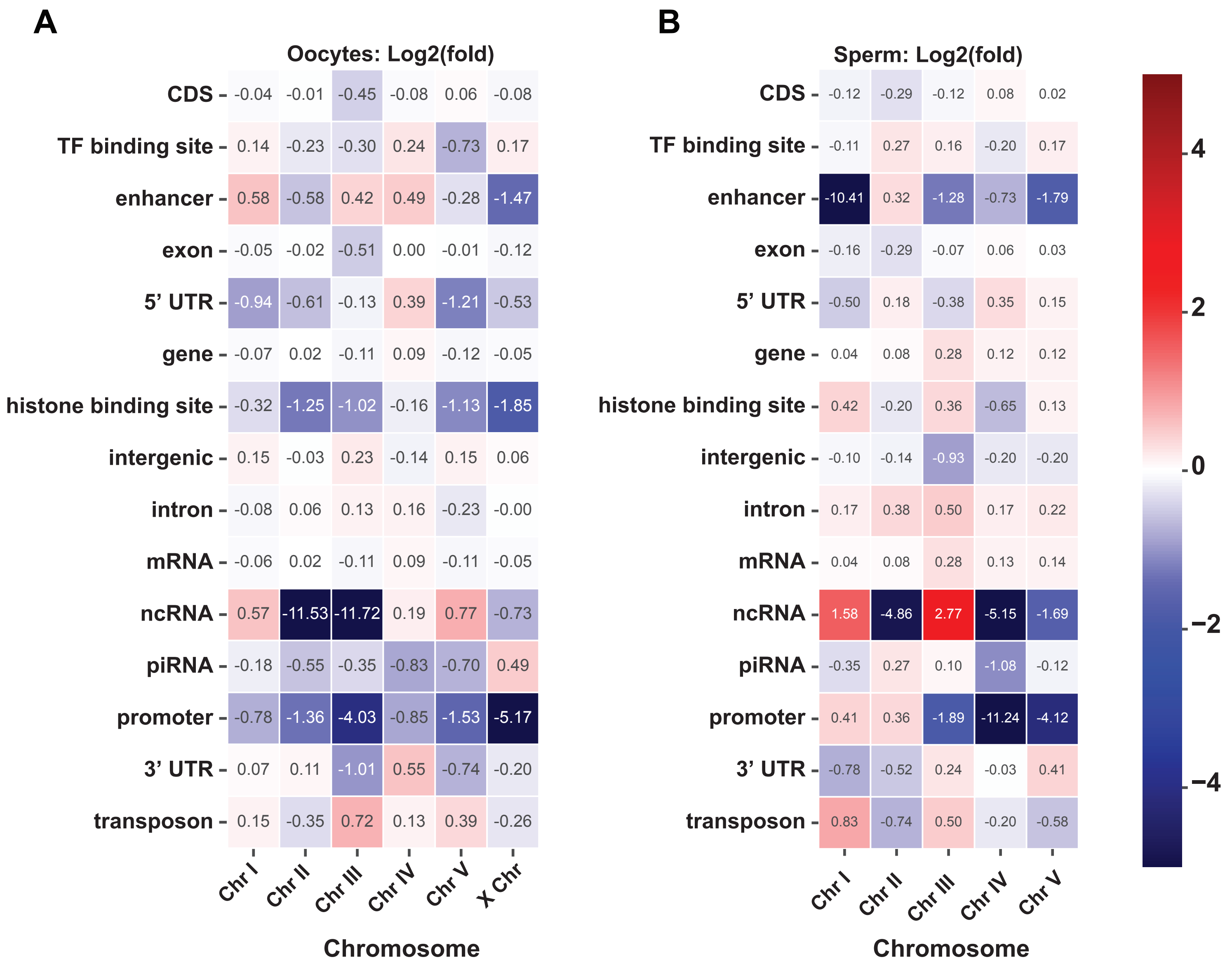

S6 Fig. Association of crossovers with sequence-level annotations. (A) Heatmap showing the log2(fold) enrichment or depletion of oocyte crossovers with each sequence annotation. (B) Heatmap showing the log2(fold) enrichment or depletion of spermatocyte crossovers with each sequence annotation. All annotations were taken from the Ensembl ce11 genome annotation set and remapped to Libuda N2 Bristol genome assembly via LiftOff (See methods).
