# Supplementary material for: High-resolution global recombination mapping in C. elegans reveals sexual dimorphisms shaped by meiotic chromosomal features and structures
Source: PLoS Genet. 2026 Jul 14;22(7):e1012237. doi: 10.1371/journal.pgen.1012237 (PMC13387615; doi:10.1371/journal.pgen.1012237)
Supplement: S7 Fig — Heatmap showing the log2(fold) association of crossovers with H3K4me3 ChIP-seq peaks. (PDF) [file pgen.1012237.s010.pdf]

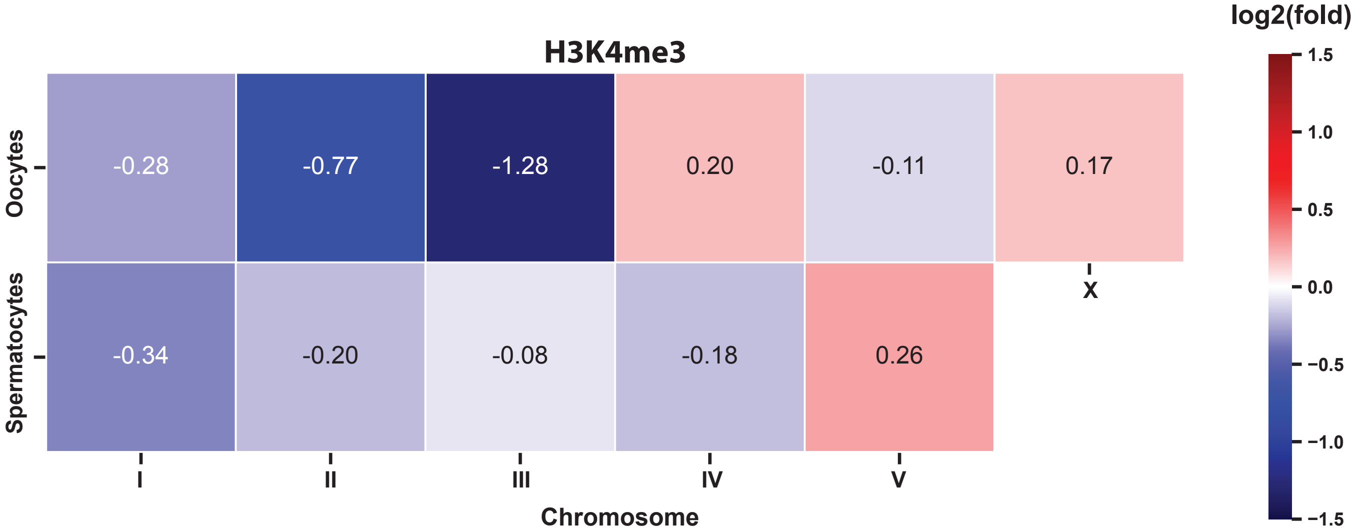

S7 Fig. Association of crossovers with H3K4me3 ChIP-seq peaks. Heatmap showing the log2(fold) association of crossovers with H3K4me3 ChIP-seq peaks.
