# Supplementary material for: High-resolution global recombination mapping in C. elegans reveals sexual dimorphisms shaped by meiotic chromosomal features and structures
Source: PLoS Genet. 2026 Jul 14;22(7):e1012237. doi: 10.1371/journal.pgen.1012237 (PMC13387615; doi:10.1371/journal.pgen.1012237)
Supplement: Attachment — Submitted filename: ResponseToReviewers.PGENETICS-D-26-00438.pdf [file pgen.1012237.s012.pdf]

We thank all of the reviewers for their thoughtful comments and critiques to further strengthen the manuscript. The incorporation of these excellent suggestions greatly improved the manuscript and its conclusions. We were excited to see that all of the reviewers recognized our manuscript as an important study determining the chromosomal structural features that shape the sexually dimorphic recombination landscape in *C. elegans*. We have addressed the essential revision comments and all the major and minor comments from the reviewers. These revisions led to the inclusion of one new figure – Supplementary Figure S7. All of our responses to individual reviewer comments can be found in BOLD in the text below. Please note that the line numbers indicated in our responses are the line numbers seen in the manuscript document with visible markup/"Track Changes".

Additional Editor Comments:

Overall, the reviewers agree that the work is interesting. Please address their concerns which can be completed through some additional analysis, statistical tests, and in the writing.

Reviewers' comments:

Reviewer's Responses to Questions

Comments to the Authors:

Reviewer #1: The manuscript by Bush et al., presents the genome-wide crossover landscape of C. elegans oocytes and spermatocytes. Leveraging WGS, the investigators show that there are significant differences in the crossover landscape on individual chromosomes and in the sexes. Analyzing a synaptonemal complex (SC) mutant heterozygote, the authors find that the SC plays a differential role in the crossover landscape on individual chromosomes and in the sexes. Chromatin environment also appears to influence the crossover landscape in a sex-specific manner. Overall, this is a well-done study that provides an important dataset to facilitate future hypotheses on crossover regulation. The following should be addressed:

Figure 1D, RAD-51 Cut + Run: It is important to state in the results that this was just done on hermaphrodites – presumably adults only undergoing oogenesis (but not in males). Given that previous published cytological and CHiP-seq data revealed enrichment of RAD-51 on chromosome arms, the authors need to discuss why their data shows a random pattern.

We appreciate the reviewer bringing up this is an important clarification, and we have added additional clarification about the use of hermaphrodite samples when first mentioned in the Results section (line 314) and Methods (lines 1032-1034)

Indeed, there is cytological evidence on computationally straightened chromosomes showing enrichment of RAD-51 on the 'arms' (Lascarez-Lagunas et al., *PLOS Genetics* 2023). The RAD-51 ChIP-seq evidence, however, is unclear. These specific RAD-51 ChIP-

seq data and the 'arm' enrichment are solely introduced in a review article (Yu *et al., Semin Cell Dev Biol.* 2017), but the plots appear illustrative and not quantitative (see screenshot below). Further, this *C. elegans* RAD-51 ChIP data, although are technically a part of the modENCODE consortium, are not included in the publicly available modENCODE datasets; therefore, we are unable to experimentally compare our dataset to this specific ChIP-seq dataset.

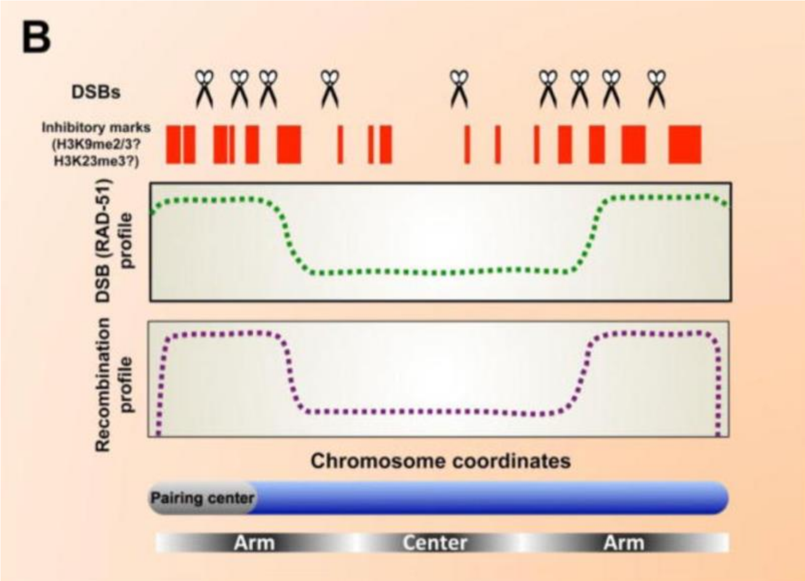

Yu *et al., Semin Cell Dev Biol.* 2017

We believe the differences in our observations and those discussed in Lascarez-Lagunas *et al.* can be attributed to the different methods employed for RAD-51 detection. Cytological quantification of RAD-51 foci along computationally straightened chromosomes operates at megabase-scale resolution, whereas our genomics approach is at sub-kilobase resolution. Further, our CUT&RUN data on bulk hermaphrodite lysate with a transient, low-abundance target like RAD-51 limits the power to detect broader, chromosome domain-level signal like 'arms' versus central enrichment. This approach and these data are powered to detect sequence-specific hotspots for DSB formation (10-100x higher than genomic background rate) but not replicate the ~3-4x enrichment across megabase-scale domains, especially after accounting for the statistical burden of testing >666,000 300 bp windows for RAD-51 binding events. Finally, cytological analysis of RAD-51 along chromosomes cannot take into account differences in chromosome compaction and DNA loop size along the axis length of the chromosome, which differ between the "arms" and "center" of the chromosome wherein gene density is much higher in the "center"; therefore the cytological assessment is a measurement of RAD-51 along an axis length as opposed to a genetic (cM) or physical length (bp) along the DNA.

To address this concern in our manuscript, we have added this context in the Results section (lines 329-342).

(Figure 1C) In the figure legend (line 1345) it states that the histogram shows global distribution of crossovers in oocytes versus oocytes – I am assuming this is supposed to be oocytes vs. spermatocytes.

We really appreciate the reviewer catching this typo. We have changed the legend text (line 1611) to accurately reflect the oocyte versus spermatocyte comparison.

Figure 3, DCOs in syp-2/+: It appears that there is no statistical difference in DCOs genome-wide in syp-2/+ oocytes and spermatocytes (Figure 3C). On individual chromosomes it appears that there are fewer DCOs on chromosome IV in oocytes and no change in spermatocytes, while an increase of DCOs on the other chromosomes. Are any of these statistically different? In the results section, please indicate that on chromosome IV, there is a reduction of DCOs in the syp-2/+ oocytes and consider the possibility that syp-2/+ alters the distribution of COs to individual chromosomes but not necessarily the number, particularly in oocytes?

In Figure 3C, we do report a significant difference in the genome-wide DCO frequency between *syp-2/+* oocytes and *syp-2/+* spermatocytes (light purple vs light green, elevated DCOs in the mutant spermatocytes). Since our statistical power is much lower for comparing the number of DCO events on individual chromosomes (*e.g.* 4 DCOs in WT oocytes vs 1 DCO in *syp-2/+* oocytes on chromosome *IV*), we want to avoid making strong claims about chromosome-specific effects in observed DCO frequencies. While the per-chromosome frequencies show qualitative variation, the most parsimonious explanation we can provide from our data is that the significant elevation in DCO frequency in both mutant eggs and sperm suggest that *syp-2/+* increases the rate of formation rather than a redistribution of events between chromosomes.

We do, however, agree that this should be noted in our results! We qualitatively discuss these interesting differences the reviewer pointed out in the Results section (lines 563-573).

Analysis of H3K36me3/H3K27me3: The difference between crossover enrichment in H3K36me3 in spermatocytes and H3K27me3 in oocytes is very interesting. However, it was unclear why only these two marks were examined. Ideally, additional euchromatin and heterochromatin marks should be examined. In the absence of this, please provide a justification for only looking at these two chromatin marks.

Thank you for returning our attention to this detail of our methods. At the time, we chose these marks/data because they were the only publicly available ChIP-seq dataset that were both germline tissue-specific and sex-specific. Other publicly available datasets did not meet these criteria for our analyses. Namely, the modENCODE data are from whole-body hermaphrodite samples, and the isolated-germ nuclei data presented in Han *et al., BMC Genomics* 2019 are solely from oocytes.

In Tabuchi *et al., Nature Communications* 2019, they isolated both spermatocytes and oocytes and performed ChIP-seq to detect H3K36me3, H3K4me3, and H3K27me3. We chose to incorporate these data into our analyses so that we could do the most accurate, germline-centric analysis, and mention this now in lines 623-625.

In Figure 4, we did not include our analysis of H3K4me3 associated with crossovers in sperm/eggs because there was no significant enrichment or depletion compared to null distributions for either sex. We now provide the H3K4me3 results in supplemental figure S7 and have added mention of this negative result in the main text (lines 634-637).

Discussion: I would really like to see a discussion of the sex-specific differences observed in this study in relation to what has been observed in other systems as indicated in the intro (lines 101-14) and perhaps what is says more globally about crossover landscapes.

The reviewer brings up a great point and something lacking in our discussion. We have added a paragraph to the discussion (lines 804-828) to provide a summary and final contextualization of our findings and how they fit within our broader understanding of crossover landscapes.

Typos:
Line 157: remove the first "the" **fixed**
Line 244: remove "the" equal **fixed**
Line 522: add "be" to "may be one" **fixed**
Line 687: remove "for" **fixed**

Reviewer #2: In this manuscript, the authors use genomic and bioinformatic analysis to analyze sexual dimorphism in crossover distribution and number during C. elegans meiosis. They show that oocytes and spermatocytes show different distributions of crossovers at large scales and finer scales, different numbers of double crossovers, and investigate whether these differences correlate with chromosomal features, such as Pairing Centers, sequence level annotations, germline gene expression and chromatin states. Moreover, they evaluate the contribution of gene dosage of a synaptonemal complex component and find that reducing syp-2 gene dosage in oocytes results in crossover distribution patterns that more closely resemble crossover distribution patterns in spermatogenesis. In general, this paper is an important contribution to the field and requires some minor revisions.

Figure 1C: I agree that oocytes show enrichment of crossovers on the PC end of Chromosomes I and III but I don't see the enrichment of crossovers on the PC end of Chromosome II. I would also include the enrichment of crossovers on the PC end of Chromosome IV but understand why they don't, given the lack of sexual dimorphism. Similarly, I agree that there are more crossovers in the central part of Chromosome V but it does not seem as dramatic as what is observed on the X chromosomes. In general. I think this section could be slightly expanded to include finer details of their analysis depicted in Figure 1.

We have expanded the detail in this section the note these observable differences in our data. This information has been added to the Results section at lines 254-260.

I appreciated the inclusion of the RAD-51 CUT&RUN data to reinforce the points about lack of hotspots.

We are delighted that the reviewer appreciates the inclusion of this important data set for the field!

Could the authors mention number of syp-2/+ genomes in results section (300 oocyte-derived and 300 spermatogenesis-derived) that make up 1292 recombinant chromosomes?

To parallel the details presented for the wild type data in the Results section, we have added this information when the *syp-2*/+ data appear in the Results section as well (lines 495-496).

Can the authors explain the lower sequencing coverage in syp-2/+ animal sequencing and if this affects their analysis?

The lower sequencing coverage per-sample is likely due to processing hundreds of our samples in a single lane on the Illumina NovaSeq platform as performed by our university's genomics core. We needed to maintain the same level of throughput and sample size for our mutant data while making it as economically feasible as possible.

From an analytical perspective, the TIGER pipeline (Rowan *et al.,* G3 2015) we have adapted for our sequencing data has been validated on real and simulated data with incredibly high accuracy at rather low sequencing coverage (97.5% accuracy at 0.1X and 99.3% at 10X coverage). For these reasons, we do not believe the lower sequencing coverage affects our analyses, and we have noted these details in the Methods section (lines 922-927) as well as noting how we did exclude several of our own samples with much lower sequencing coverage than what was used as validation for the TIGER pipeline (lines 1004-1006).

For those not well-versed in these approaches to assess crossover number and distribution: Is there any concern about bias in what fraction of chromosomes are reported for crossover location and number?

We do not believe there is concern about the fraction of recombinant calls per chromosome within experimental groups. Excluding calls on the X chromosome (because of the hemizygosity in spermatocytes), there is <10% difference in the total number of recombinant calls across autosomes between sperm and eggs for the wild type and *syp-2*/+ mutant datasets (Tables 1 and 2). While there is slightly higher variance in per-chromosome calls, this can largely be explained by random segregation variance expected from a finite sampling of ~300 individuals in each experimental group. We also highlight other factors that make minor contributions to this variance such as a greater number of low-coverage spermatocyte samples and known genetic incompatibilities (the *peel-1 zeel-1* toxin-antidote system) between our Bristol and Hawaiian parental strains in the results (lines 217-226).

Could the authors specify in their analysis of chromatin states and crossover distributions that the histone modification data comes from analysis of germ cells?

We agree with you and Reviewer 1 that this detail is important for our germline-centric analysis, and we have added this information at lines 623-625 in the Results section.

In their section of the discussion, “Chromosome synapsis and sexually dimorphic crossover distributions,” the authors discuss synapsis defects as a possible source of the altered crossover distributions in syp-2/+ oocytes. Another possibility the authors should consider discussing is that SYP-2 dosage in syp-2/+ oocytes approximates SYP-2 dosage in wildtype spermatogenesis. For example, Cahoon et al show that SYP-2 fluorescence on meiotic chromosomes is reduced at early stages of meiosis during spermatogenesis, compared to oogenesis. Given their analysis that syp-2 gene dosage affects crossover distributions and number in both sexes, it’s possible that the amount of SYP-2 in the synaptonemal complex contributes to control of these events. Similarly, syp-2 gene dosage could have consequences on the amount of other synaptonemal complex components in this structure, affecting crossover distribution aside from affecting the process of synapsis itself.

This is an incredibly thoughtful point that we did not originally consider, and we thank you for bringing this to light. We have added the information surrounding this hypothesis to our Discussion section (lines 707-715)

In lines 521-523, the be is missing: the prolonged duration of pairing in wild-type C. elegans oogenesis may *be* one factor that contributes to the broad-scale differences in the crossover distribution on the pairing center “arms” of these chromosomes.

Fixed!

Reviewer #3: Bush et al. used whole-genome sequencing to map crossovers and non-crossover gene conversion events in C. elegans males and females. Intensive analysis of the sequencing data provides support for several conclusions. Although some of these may seem to merely buttress results from genetic experiments, these have typically been restricted to part of a single chromosome pair. The genome-wide analyses in this manuscript provide robust data along each chromosome, among chromosomes, and between the sexes. This and the accompanying analyses (e.g., the SC mutant) make a significant contribution to quantifying differences in crossover distribution with respect to several metrics. Although most of the manuscript focuses on crossover positions, mapping of non-crossovers and of RAD-51 binding bring additional important insights.

The analysis is thorough and broad. I don’t believe there are additional experiments that need to be done, but the points below should be addressed in revision.

1. RAD-51 binding sites are found across every chromosome, including in the central region of

each, so one might expect total recombination frequency (NCO + CO) to mirror this. The lower SNP density in the central regions means that if there are COs there they will be mapped with low resolution, but still detected. As noted, NCOs in these regions will be missed because of low SNP density. The regions of low SNP density seem to be a third to a half of each chromosome. This makes it puzzling that the total NCOs observed is approximately the number predicted through other studies (lines 245-257). Can the authors reconcile these observations?

We thank the reviewer for pointing out this confounding point in our manuscript. The total number of DSBs per nucleus has been highly contested and may be quite variable. All estimates of DSBs per nucleus, to our knowledge, stem for cytological analysis of RAD-51 foci per nucleus, and the most recent estimates in *C. elegans* suggest that there may be between 20-38 RAD-51 per nucleus (Hamrick et al., *Genetics* 2026). The authors of that study also claim they are likely underestimating this count for a number of reasons. If a nucleus incurs something around the midpoint of this range at ~29 DSBs per nucleus, then there may be ~23 NCOs per nucleus after 6 crossovers (largely 1 per chromosome) are accounted for.

By stating we are able to detect 17-18 per genome, and we admit we are likely undercounting as well due to the limits of our detection methods across the center of each chromosome. We have dialed back our verbiage in that section and added clarification to indicate that we don't expect to account for all DSBs incurred per nucleus, purely that we do not see an excess of NCO calls that would suggest the total number of recombination events per nucleus exceeds what we expect for the average amount of preceding DSBs (lines 293-307).

We also acknowledge the gap between what our methods and cytological studies detect for RAD-51 sites and recombination events in our response to Reviewer 1:

We believe the differences in our observations and those discussed in Lascarez-Lagunas *et al.* can be attributed to the different methods employed for RAD-51 detection. Cytological quantification of RAD-51 foci along computationally straightened chromosomes operates at megabase-scale resolution, whereas our genomics approach is at sub-kilobase resolution. Further, our CUT&RUN data on bulk hermaphrodite lysate with a transient, low-abundance target like RAD-51 limits the power to detect broader, chromosome domain-level signal like 'arms' versus central enrichment. This approach and these data are powered to detect sequence-specific hotspots for DSB formation (10-100x higher than genomic background rate) but not replicate the ~3-4x enrichment across megabase-scale domains, especially after accounting for the statistical burden of testing >666,000 300 bp windows for RAD-51 binding events. Finally, cytological analysis of RAD-51 along chromosomes cannot take into account differences in chromosome compaction and DNA loop size along the axis length of the chromosome, which differ between the "arms" and "center" of the chromosome wherein gene density is much higher in the "center"; therefore the cytological assessment is a measurement of RAD-51 along an axis length as opposed to a genetic (cM) or physical length (bp) along the DNA.

To address this concern in our manuscript, we have added this context in the Results section (lines 329-342).

2. The heatmaps in Figs 3 and 4 can be difficult to parse. There are certainly some features that

must be analyzed on a per chromosome basis, but is there any reason to expect that association with chromatin or gene expression states would vary between chromosomes (or perhaps between groups of chromosomes that seem to be similar, i.e. [I, II, & III], [IV, V], [X])?

We share some of the reviewer's impressions about the complexities to p-value heatmap visualizations in Figure 3 and have had much debate within our lab and among data science colleagues on how to best visualize these multi-comparative statistical analyses. Our goal is to show the WT and mutant crossover frequency maps simultaneously to aid visual comparison across all four experimental groups. To include indicators of statistical significance (*e.g.* asterisks, brackets, etc.) on top of these often-intersecting/overlapping lines would add too much complexity to an already busy visualization. We also considered including a table for each pairwise comparison so readers could track statistical significance in that format, but the table would be quite long (30 rows) and potentially occupy a whole page or merely fall into supplement (which would not benefit a reader trying to parse out conclusions from the main figures).

For Figure 4, we did try aggregating results and performing a genome-wide analysis of association between crossover and chromatin states, for example. In this analysis, the effect sizes were incredibly small and/or statistically insignificant. The chromosome-level resolution in this analysis is needed to capture biological differences between the sexes. In our analysis to identify a sexual dimorphism, the *X* chromosome is always excluded due to the hemizygosity in males/sperm. We have tried grouping certain autosomes together but eventually decided that it didn't add any significant conclusions to the data. Also, we were unable to find this type *ad hoc* analytical grouping of chromosomes in our review of the other published recombination studies in *C. elegans*.

3. Tables 1 and 2 should include number of chromosomes sequenced, as well as an indication of what is statistically significant (most informative might be oocyte versus spermatocyte).

We appreciate the reviewer's suggestion but we think it will be visually redundant to include the number of sequenced chromosomes in these tables. We indicate the total number of samples sequenced in the results (lines 203, 209-211, and 495-496) and Methods (lines 883-886). This would result in entire rows filled by the same 300 or 310 values, and we provide criteria/discussion for why samples may be excluded from data analysis (*e.g.* insufficient read data, lines 1004-1006). Moreover, since we are not trying to make statistical claims about the number of chromosomes called as having crossovers, this addition would not address any of the biological claims we make in the manuscript.

4. In several places, the authors use logistic regression analyses, but there are no details provided. For example, is the model being used binomial (e.g., parental versus recombinant) or multinomial (parental, single crossover, double crossover)?

We thank the reviewer for pointing out this lack of clarity on our logistic regression analyses. For all analyses where we used logistic regression, there is a binary response variable. For our analyses of DCO frequencies, this is merely is/is not a DCO event for each predictor group. For our analyses of COs on the PC-bearing chromosome arms, the response variable was also a binary is/is not on the PC arm. We have now added the

clarification that our logistic regression models are binomial in nature at each mention in the text. We have also added specific clarification in the Methods section (lines 1096-1104)

Typos and minor corrections:

• line 81: phenomenon is the singular

Fixed.

• line 151: “near sites where homologous chromosomes pair” should instead say where pairing is initiated, since they eventually are paired along their entire lengths

Thank you for catching this, we have changed the text to reflect this accuracy.

• lines 191-194: The wording is confusing. Does “…our crossover mapping pipeline was able to successfully call crossovers in 297/300 oocyte samples and 300/310 spermatocyte samples” mean the pipeline failed for 3 oocyte and 10 spermatocyte samples or that there were just no crossovers detected in those samples? The numbers are about what would be expected if there is an average of one crossover per bivalent and recovery of nonrecombinant versus recombinant chromatids is independent between bivalents. Also, since the first clause is “In the oocyte data,” the last clause “and 738…” implies that the spermatocyte data are from the oocyte data.

This means that for those 12 samples, no crossovers were called due to insufficient read coverage, and this detail is in our Methods section (lines 1004-1006). We have also clarified the text around numbers of crossovers-bearing chromosomes detected on lines 211-213.

• lines 198-203: This paragraph concludes by saying that the whole-genome sequence method is reliable, but it’s unclear how the preceding two sentences support this conclusion. Rather, it appears that the intent is to say that despite some samples having insufficient read coverage (as is to be expected) and there being genetic incompatibilities), this approach is still informative. Is the argument that because there are distinctive patterns detected across multiple analyses (examples should be given or something like “described below” included) this lends validity to the approach?

We appreciate learning about the position contradictory nature of our rhetoric. We were trying to address the variance in number of chromosomes crossovers detected by our methods. We have now modified the text at lines 217-227 to be more in line with our intent.

• lines 210-213: The sex-specific bias is noted for I, II, and III, but later IV is lumped in as being different from V and X.

We have added clarity about this sexual-dimorphism (or lack-thereof on chromosome IV) at lines 254-256.

• lines 214-215: What does "seemingly elevated" mean? Is there a statistically significant elevation?

We did not perform a separate statistical test specifically for the proportion crossovers in central versus arm domains. We have added clarity at lines 256-260 that the observation is merely qualitative.

• line 260-262: It's not really "base pair resolution" obtained with CUT&RUN and 300 bp windows. Also, the analyses cannot reveal that there are "no statistically enriched binding sites for RAD-51," only that none were detected with the parameters used.

We have amended this to "sub-kilobase resolution" (line 332) and clarified that we did not detect enriched binding sites given our parameters to avoid confusion (lines 333-335).

• line 265: The suggestion of peaks of RAD-51 about every 10 kb is interesting. Does this correspond to any known or hypothesized structural feature of meiotic chromosomes?

We agree that this potential 10 kb periodicity of the RAD-51 peaks is interesting, which is why we wanted to include this observation in the text. Unfortunately, to our knowledge, this does not correlate with any structural features specific to meiotic chromosomes in *C. elegans.*

• line 290: "each" should be "one"

Fixed.

• lines 291-301: The apparent sexually dimorphic windows may be interesting, but the discussion lacks some statistical framework. For the analysis, what is the interval between sliding windows, and how many such windows were compared in total? Figure 2B shows the sex-specific differences clearly, but the numbers seen here appear to be far lower than stated in the text (e.g., it doesn't look like there are 31 adjacent differences for chromosome I). Is this because the figure shows only one set of adjacent 200 kb intervals, with no sliding?

For all sliding window analyses as depicted in Figure 2B (and related Supplemental Figure S4), the step size/overlap for successive windows is 50% of the window size. This detail is in the legend for Figure 2 (line 1627), but we have also added more detail in the

Results section at lines 389-390. For Figure 2, there are 820 total 200 kb windows created by our sliding window parameters, and all 820 windows are represented in the line plot, though many have a log2 value of zero. To add clarity, we have also adopted the usage of "successive windows" to avoid confusion on the overlap/adjacency of windows, as successive windows do overlap by 50%.

As for the number of windows where successive windows display changes in which sex has the highest crossover rate, we did catch a coding error in the function that counted such occurrences. This error overestimated 'switching' counts by also including changes from equal-rate windows to windows where the rate of one sex dominates. Thank you for bringing greater attention to this data. Our larger conclusions do not change, but we have amended the counts in the appropriate results text (lines 390-400).

• line 352-353: "is sexually dimorphic…in oocytes versus spermatocytes" is redundant

Fixed.

• lines 353-355: "…and some of this sexual dimorphism can be attributed to crossover formation relative to their distance from the chromosome pairing centers." The phrase "can be attributed to" suggest that the distance is the cause of the dimorphism. It may be, but the data presented only show that this is an observation. The observation is that a greater proportion of oocyte crossovers are within or near the PCs.

We agree this was not the framing we wanted to achieve with this concluding sentence. We have amended this text at lines 465-480.

• line 616: "during in"

Fixed.

• The term "arm" is sometimes in quotation marks and sometimes not. For consistency, I suggest that early in the manuscript the authors clearly define what they are going to refer to as an arm (e.g., a percentage of physical or genetic length or the region with high SNP density) and then drop the quotation marks.

We have removed the excessive/inconsistent usage of quotation marks. We have briefly describe how we define a chromosome "arm" in *C. elegans* at first mention in the results (lines 237-241) and Methods (lines 1087-1095).

• All of the intervals are defined by SNPs. One presumes there are also indels. Were these not used because of mapping complications?

Correct, indels were not used for recombination mapping. The indels introduce potential inaccuracies in read mapping that could skew the identification of heterozygous sites needed for breakpoint resolution. A comment on this has been added at lines 897-899.

• line 719: Does accuracy refer to call v. not called or precision of the location? Even at 0.1x coverage it seems like very crossover should be called, just with less precision on the position.

For the TIGER pipeline, accuracy does indeed refer to call vs not-called across the validated positions. The precision of this method calls crossovers within 2 kb at all tested coverage levels (0.1X, 1X, and 10X). We have added more of this information at lines 924-927.
