# Supplementary material for: High-resolution global recombination mapping in C. elegans reveals sexual dimorphisms shaped by meiotic chromosomal features and structures
Source: PLoS Genet. 2026 Jul 14;22(7):e1012237. doi: 10.1371/journal.pgen.1012237 (PMC13387615; doi:10.1371/journal.pgen.1012237)
Supplement: Peer review history

# Peer review: Decision Letter 0

**DOI:** 10.1371/journal.pgen.1012237.r001

**Authors:** Marnie E. Blewitt, Soni Lacefield

26 May 2026

PGENETICS-D-26-00438

High-resolution global recombination mapping in C. elegans reveals sexual dimorphisms shaped by meiotic chromosomal features and structures

PLOS Genetics

Dear Dr. Libuda,

Thank you for submitting your manuscript to PLOS Genetics. After careful consideration, we feel that it has merit but does not fully meet PLOS Genetics's publication criteria as it currently stands. Therefore, we invite you to submit a revised version of the manuscript that addresses the points raised during the review process.

Please submit your revised manuscript within by Jun 25 2026 11:59PM. If you will need more time than this to complete your revisions, please reply to this message or contact the journal office at plosgenetics@plos.org. When you're ready to submit your revision, log on to https://www.editorialmanager.com/pgenetics/ and select the 'Submissions Needing Revision' folder to locate your manuscript file.

Please include the following items when submitting your revised manuscript:

* A letter that responds to each point raised by the editor and reviewer(s). You should upload this letter as a separate file labeled 'Response to Reviewers'. This file does not need to include responses to formatting updates and technical items listed in the 'Journal Requirements' section below.

* A marked-up copy of your manuscript that highlights changes made to the original version. You should upload this as a separate file labeled 'Revised Manuscript with Track Changes'.

* An unmarked version of your revised paper without tracked changes. You should upload this as a separate file labeled 'Manuscript'.

If you would like to make changes to your financial disclosure, competing interests statement, or data availability statement, please make these updates within the submission form at the time of resubmission. Guidelines for resubmitting your figure files are available below the reviewer comments at the end of this letter.

As the corresponding author, your ORCID iD is verified in the submission system and will appear in the published article. PLOS supports the use of ORCID, and we encourage all coauthors to register for an ORCID iD and use it as well. Please encourage your coauthors to verify their ORCID iD within the submission system before final acceptance, as unverified ORCID iDs will not appear in the published article. Only the individual author can complete the verification step; PLOS staff cannot verify ORCID iDs on behalf of authors.

We look forward to receiving your revised manuscript.

Kind regards,

Soni Lacefield, PhD

Academic Editor

PLOS Genetics

Marnie Blewitt

Section Editor

PLOS Genetics

Aimée Dudley

Editor-in-Chief

PLOS Genetics

Anne Goriely

Editor-in-Chief

PLOS Genetics

**Additional Editor Comments:**

Overall, the reviewers agree that the work is interesting. Please address their concerns which can be completed through some additional analysis, statistical tests, and in the writing.

**Journal Requirements:**

If the reviewer comments include a recommendation to cite specific previously published works, please review and evaluate these publications to determine whether they are relevant and should be cited. There is no requirement to cite these works unless the editor has indicated otherwise.

1) We ask that a manuscript source file is provided at Revision. Please upload your manuscript file as a .doc, .docx, .rtf or .tex. If you are providing a .tex file, please upload it under the item type u2018LaTeX Source Fileu2019 and leave your .pdf version as the item type u2018Manuscriptu2019.

2) We do not publish any copyright or trademark symbols that usually accompany proprietary names, eg ©,  ®, or TM  (e.g. next to drug or reagent names). Therefore please remove all instances of trademark/copyright symbols throughout the text, including:

- TM on pages: 27, and 33.

3) Please upload all main figures as separate Figure files in .tif or .eps format. For more information about how to convert and format your figure files please see our guidelines:

https://journals.plos.org/plosgenetics/s/figures

4) We notice that your supplementary Figures are included in the manuscript file. Please remove them and upload them with the file type 'Supporting Information'. Please ensure that each Supporting Information file has a legend listed in the manuscript after the references list.

5) Some material included in your submission may be copyrighted. According to PLOS’s copyright policy, authors who use figures or other material (e.g., graphics, clipart, maps) from another author or copyright holder must demonstrate or obtain permission to publish this material under the Creative Commons Attribution 4.0 International (CC BY 4.0) License used by PLOS journals. Please closely review the details of PLOS’s copyright requirements here: PLOS Licenses and Copyright. If you need to request permissions from a copyright holder, you may use PLOS's Copyright Content Permission form.

Please respond directly to this email and provide any known details concerning your material's license terms and permissions required for reuse, even if you have not yet obtained copyright permissions or are unsure of your material's copyright compatibility. Once you have responded and addressed all other outstanding technical requirements, you may resubmit your manuscript within Editorial Manager.

Potential Copyright Issues:

i) Figures 1A, and S2B. Please confirm whether you drew the images / clip-art within the figure panels by hand. If you did not draw the images, please provide (a) a link to the source of the images or icons and their license / terms of use; or (b) written permission from the copyright holder to publish the images or icons under our CC BY 4.0 license. Alternatively, you may replace the images with open source alternatives. See these open source resources you may use to replace images / clip-art:

- https://commons.wikimedia.org

- https://openclipart.org/.

6) When completing the data availability statement of the submission form, you indicated that you will make your data available on acceptance. We strongly recommend all authors decide on a data sharing plan before acceptance, as the process can be lengthy and hold up publication timelines. Please note that, though access restrictions are acceptable now, your entire data will need to be made freely accessible if your manuscript is accepted for publication. This policy applies to all data except where public deposition would breach compliance with the protocol approved by your research ethics board. If you are unable to adhere to our open data policy, please kindly revise your statement to explain your reasoning and we will seek the editor's input on an exemption. Please be assured that, once you have provided your new statement, the assessment of your exemption will not hold up the peer review process.

7) Please amend your detailed Financial Disclosure statement. This is published with the article. It must therefore be completed in full sentences and contain the exact wording you wish to be published.

1) State what role the funders took in the study. If the funders had no role in your study, please state: "The funders had no role in study design, data collection and analysis, decision to publish, or preparation of the manuscript."

2) If any authors received a salary from any of your funders, please state which authors and which funders..

If you did not receive any funding for this study, please simply state: u201cThe authors received no specific funding for this work.u201d

8)  Please ensure that the funders and grant numbers match between the Financial Disclosure field and the Funding Information tab in your submission form. Note that the funders must be provided in the same order in both places as well.

**Reviewers' comments:**

Reviewer's Responses to Questions

**Comments to the Authors:**

**Please note here if the review is uploaded as an attachment.**

Reviewer #1: The manuscript by Bush et al., presents the genome-wide crossover landscape of C. elegans oocytes and spermatocytes. Leveraging WGS, the investigators show that there are significant differences in the crossover landscape on individual chromosomes and in the sexes. Analyzing a synaptonemal complex (SC) mutant heterozygote, the authors find that the SC plays a differential role in the crossover landscape on individual chromosomes and in the sexes. Chromatin environment also appears to influence the crossover landscape in a sex-specific manner. Overall, this is a well-done study that provides an important dataset to facilitate future hypotheses on crossover regulation. The following should be addressed:

Figure 1D, RAD-51 Cut + Run: It is important to state in the results that this was just done on hermaphrodites – presumably adults only undergoing oogenesis (but not in males). Given that previous published cytological and CHiP-seq data revealed enrichment of RAD-51 on chromosome arms, the authors need to discuss why their data shows a random pattern. In the figure legend (line 1345) it states that the histogram shows global distribution of crossovers in oocytes versus oocytes – I am assuming this is supposed to be oocytes vs. spermatocytes.

Figure 3, DCOs in syp-2/+: It appears that there is no statistical difference in DCOs genome-wide in syp-2/+ oocytes and spermatocytes (Figure 3C). On individual chromosomes it appears that there are fewer DCOs on chromosome IV in oocytes and no change in spermatocytes, while an increase of DCOs on the other chromosomes. Are any of these statistically different? In the results section, please indicate that on chromosome IV, there is a reduction of DCOs in the syp-2/+ oocytes and consider the possibility that syp-2/+ alters the distribution of COs to individual chromosomes but not necessarily the number, particularly in oocytes?

Analysis of H3K36me3/H3K27me3: The difference between crossover enrichment in H3K36me3 in spermatocytes and H3K27me3 in oocytes is very interesting. However, it was unclear why only these two marks were examined. Ideally, additional euchromatin and heterochromatin marks should be examined. In the absence of this, please provide a justification for only looking at these two chromatin marks.

Discussion: I would really like to see a discussion of the sex-specific differences observed in this study in relation to what has been observed in other systems as indicated in the intro (lines 101-14) and perhaps what is says more globally about crossover landscapes.

Typos:

Line 157: remove the first “the”

Line 244: remove “the” equal

Line 522: add “be” to “may be one”

Line 687: remove “for”

Reviewer #2: In this manuscript, the authors use genomic and bioinformatic analysis to analyze sexual dimorphism in crossover distribution and number during C. elegans meiosis. They show that oocytes and spermatocytes show different distributions of crossovers at large scales and finer scales, different numbers of double crossovers, and investigate whether these differences correlate with chromosomal features, such as Pairing Centers, sequence level annotations, germline gene expression and chromatin states. Moreover, they evaluate the contribution of gene dosage of a synaptonemal complex component and find that reducing syp-2 gene dosage in oocytes results in crossover distribution patterns that more closely resemble crossover distribution patterns in spermatogenesis. In general, this paper is an important contribution to the field and requires some minor revisions.

Figure 1C: I agree that oocytes show enrichment of crossovers on the PC end of Chromosomes I and III but I don’t see the enrichment of crossovers on the PC end of Chromosome II. I would also include the enrichment of crossovers on the PC end of Chromosome IV but understand why they don’t, given the lack of sexual dimorphism. Similarly, I agree that there are more crossovers in the central part of Chromosome V but it does not seem as dramatic as what is observed on the X chromosomes. In general. I think this section could be slightly expanded to include finer details of their analysis depicted in Figure 1.

I appreciated the inclusion of the RAD-51 CUT&RUN data to reinforce the points about lack of hotspots.

Could the authors mention number of syp-2/+ genomes in results section (300 oocyte-derived and 300 spermatogenesis-derived) that make up 1292 recombinant chromosomes?

Can the authors explain the lower sequencing coverage in syp-2/+ animal sequencing and if this affects their analysis?

For those not well-versed in these approaches to assess crossover number and distribution: Is there any concern about bias in what fraction of chromosomes are reported for crossover location and number?

Could the authors specify in their analysis of chromatin states and crossover distributions that the histone modification data comes from analysis of germ cells?

In their section of the discussion, “Chromosome synapsis and sexually dimorphic crossover distributions,” the authors discuss synapsis defects as a possible source of the altered crossover distributions in syp-2/+ oocytes. Another possibility the authors should consider discussing is that SYP-2 dosage in syp-2/+ oocytes approximates SYP-2 dosage in wildtype spermatogenesis. For example, Cahoon et al show that SYP-2 fluorescence on meiotic chromosomes is reduced at early stages of meiosis during spermatogenesis, compared to oogenesis. Given their analysis that syp-2 gene dosage affects crossover distributions and number in both sexes, it’s possible that the amount of SYP-2 in the synaptonemal complex contributes to control of these events. Similarly, syp-2 gene dosage could have consequences on the amount of other synaptonemal complex components in this structure, affecting crossover distribution aside from affecting the process of synapsis itself.

In lines 521-523, the be is missing: the prolonged duration of pairing in wild-type C. elegans oogenesis may *be* one factor that contributes to the broad-scale differences in the crossover distribution on the pairing center “arms” of these chromosomes.

Reviewer #3: Bush et al. used whole-genome sequencing to map crossovers and non-crossover gene conversion events in C. elegans males and females. Intensive analysis of the sequencing data provides support for several conclusions. Although some of these may seem to merely buttress results from genetic experiments, these have typically been restricted to part of a single chromosome pair. The genome-wide analyses in this manuscript provide robust data along each chromosome, among chromosomes, and between the sexes. This and the accompanying analyses (e.g., the SC mutant) make a significant contribution to quantifying differences in crossover distribution with respect to several metrics. Although most of the manuscript focuses on crossover positions, mapping of non-crossovers and of RAD-51 binding bring additional important insights.

The analysis is thorough and broad. I don’t believe there are additional experiments that need to be done, but the points below should be addressed in revision.

1. RAD-51 binding sites are found across every chromosome, including in the central region of each, so one might expect total recombination frequency (NCO + CO) to mirror this. The lower SNP density in the central regions means that if there are COs there they will be mapped with low resolution, but still detected. As noted, NCOs in these regions will be missed because of low SNP density. The regions of low SNP density seem to be a third to a half of each chromosome. This makes it puzzling that the total NCOs observed is approximately the number predicted through other studies (lines 245-257). Can the authors reconcile these observations?

2. The heatmaps in Figs 3 and 4 can be difficult to parse. There are certainly some features that must be analyzed on a per chromosome basis, but is there any reason to expect that association with chromatin or gene expression states would vary between chromosomes (or perhaps between groups of chromosomes that seem to be similar, i.e. [I, II, & III], [IV, V], [X])?

3. Tables 1 and 2 should include number of chromosomes sequenced, as well as an indication of what is statistically significant (most informative might be oocyte versus spermatocyte).

4. In several places, the authors use logistic regression analyses, but there are no details provided. For example, is the model being used binomial (e.g., parental versus recombinant) or multinomial (parental, single crossover, double crossover)?

Typos and minor corrections:

• line 81: phenomenon is the singular

• line 151: “near sites where homologous chromosomes pair” should instead say where pairing is initiated, since they eventually are paired along their entire lengths

• lines 191-194: The wording is confusing. Does “…our crossover mapping pipeline was able to successfully call crossovers in 297/300 oocyte samples and 300/310 spermatocyte samples” mean the pipeline failed for 3 oocyte and 10 spermatocyte samples or that there were just no crossovers detected in those samples? The numbers are about what would be expected if there is an average of one crossover per bivalent and recovery of nonrecombinant versus recombinant chromatids is independent between bivalents. Also, since the first clause is “In the oocyte data,” the last clause “and 738…” implies that the spermatocyte data are from the oocyte data.

• lines 198-203: This paragraph concludes by saying that the whole-genome sequence method is reliable, but it’s unclear how the preceding two sentences support this conclusion. Rather, it appears that the intent is to say that despite some samples having insufficient read coverage (as is to be expected) and there being genetic incompatibilities), this approach is still informative. Is the argument that because there are distinctive patterns detected across multiple analyses (examples should be given or something like “described below” included) this lends validity to the approach?

• lines 210-213: The sex-specific bias is noted for I, II, and III, but later IV is lumped in as being different from V and X.

• lines 214-215: What does “seemingly elevated” mean? Is there a statistically significant elevation?

• line 260-262: It’s not really “base pair resolution” obtained with CUT&RUN and 300 bp windows. Also, the analyses cannot reveal that there are “no statistically enriched binding sites for RAD-51,” only that none were detected with the parameters used.

• line 265: The suggestion of peaks of RAD-51 about every 10 kb is interesting. Does this correspond to any known or hypothesized structural feature of meiotic chromosomes?

• line 290: “each” should be “one”

• lines 291-301: The apparent sexually dimorphic windows may be interesting, but the discussion lacks some statistical framework. For the analysis, what is the interval between sliding windows, and how many such windows were compared in total? Figure 2B shows the sex-specific differences clearly, but the numbers seen here appear to be far lower than stated in the text (e.g., it doesn’t look like there are 31 adjacent differences for chromosome I). Is this because the figure shows only one set of adjacent 200 kb intervals, with no sliding?

• line 352-353: “is sexually dimorphic…in oocytes versus spermatocytes” is redundant

• lines 353-355: “…and some of this sexual dimorphism can be attributed to crossover formation relative to their distance from the chromosome pairing centers.” The phrase “can be attributed to” suggest that the distance is the cause of the dimorphism. It may be, but the data presented only show that this is an observation. The observation is that a greater proportion of oocyte crossovers are within or near the PCs.

• line 616: “during in”

• The term “arm” is sometimes in quotation marks and sometimes not. For consistency, I suggest that early in the manuscript the authors clearly define what they are going to refer to as an arm (e.g., a percentage of physical or genetic length or the region with high SNP density) and then drop the quotation marks.

• All of the intervals are defined by SNPs. One presumes there are also indels. Were these not used because of mapping complications?

• line 719: Does accuracy refer to call v. not called or precision of the location? Even at 0.1x coverage it seems like very crossover should be called, just with less precision on the position.

**********

**Have all data underlying the figures and results presented in the manuscript been provided?**

Large-scale datasets should be made available via a public repository as described in the *PLOS Genetics*  data availability policy, and numerical data that underlies graphs or summary statistics should be provided in spreadsheet form as supporting information.

Reviewer #1: Yes

Reviewer #2: **No:** Illumina data availability is currently in progress and I didn't see numerical data for the graphs.

Reviewer #3: Yes

**********

PLOS authors have the option to publish the peer review history of their article (what does this mean?). If published, this will include your full peer review and any attached files.

If you choose “no”, your identity will remain anonymous but your review may still be made public.

**Do you want your identity to be public for this peer review?**  For information about this choice, including consent withdrawal, please see our Privacy Policy.

Reviewer #1: No

Reviewer #2: No

Reviewer #3: No

**Figure resubmission:**

While revising your submission, we strongly recommend that you use PLOS’s NAAS tool (https://ngplosjournals.pagemajik.ai/artanalysis) to test your figure files. NAAS can convert your figure files to the TIFF file type and meet basic requirements (such as print size, resolution), or provide you with a report on issues that do not meet our requirements and that NAAS cannot fix.

After uploading your figures to PLOS’s NAAS tool - https://ngplosjournals.pagemajik.ai/artanalysis, NAAS will process the files provided and display the results in the "Uploaded Files" section of the page as the processing is complete. If the uploaded figures meet our requirements (or NAAS is able to fix the files to meet our requirements), the figure will be marked as "fixed" above. If NAAS is unable to fix the files, a red "failed" label will appear above. When NAAS has confirmed that the figure files meet our requirements, please download the file via the download option, and include these NAAS processed figure files when submitting your revised manuscript.

**Reproducibility:**

To enhance the reproducibility of your results, we recommend that authors deposit laboratory protocols in protocols.io, where a protocol can be assigned its own identifier (DOI) such that it can be cited independently in the future. Additionally, PLOS ONE offers an option to publish peer-reviewed clinical study protocols. Read more information on sharing protocols at https://plos.org/protocols?utm_medium=editorial-email&utm_source=authorletters&utm_campaign=protocols

# Author comment: Author response to Decision Letter 1

**DOI:** 10.1371/journal.pgen.1012237.r002

26 Jun 2026

Attachment Submitted filename: ResponseToReviewers.PGENETICS-D-26-00438.pdf

# Editor report: Decision Letter 1

**DOI:** 10.1371/journal.pgen.1012237.r003

**Authors:** Marnie E. Blewitt, Soni Lacefield

4 Jul 2026

Dear Dr Libuda,

We are pleased to inform you that your manuscript entitled "High-resolution global recombination mapping in C. elegans reveals sexual dimorphisms shaped by meiotic chromosomal features and structures" has been editorially accepted for publication in PLOS Genetics. Congratulations!

Before your submission can be formally accepted and sent to production you will need to complete our formatting changes, which you will receive in a follow up email. Please be aware that it may take several days for you to receive this email; during this time no action is required by you. Please note: the accept date on your published article will reflect the date of this provisional acceptance, but your manuscript will not be scheduled for publication until the required changes have been made.

Once your paper is formally accepted, an uncorrected proof of your manuscript will be published online ahead of the final version, unless you’ve already opted out via the online submission form. If, for any reason, you do not want an earlier version of your manuscript published online or are unsure if you have already indicated as such, please let the journal staff know immediately at plosgenetics@plos.org.

In the meantime, please log into Editorial Manager at https://www.editorialmanager.com/pgenetics/, click the "Update My Information" link at the top of the page, and update your user information to ensure an efficient production and billing process. Note that PLOS requires an ORCID iD for all corresponding authors. Therefore, please ensure that you have an ORCID iD and that it is validated in Editorial Manager. To do this, go to ‘Update my Information’ (in the upper left-hand corner of the main menu), and click on the Fetch/Validate link next to the ORCID field.  This will take you to the ORCID site and allow you to create a new iD or authenticate a pre-existing iD in Editorial Manager.

If you have a press-related query, or would like to know about making your underlying data available (as you will be aware, this is required for publication), please see the end of this email. If your institution or institutions have a press office, please notify them about your upcoming article at this point, to enable them to help maximise its impact. Inform journal staff as soon as possible if you are preparing a press release for your article and need a publication date.

Thank you again for supporting open-access publishing; we are looking forward to publishing your work in PLOS Genetics!

Yours sincerely,

Soni Lacefield, PhD

Academic Editor

PLOS Genetics

Marnie Blewitt

Section Editor

PLOS Genetics

Aimée Dudley

Editor-in-Chief

PLOS Genetics

Anne Goriely

Editor-in-Chief

PLOS Genetics

www.plosgenetics.org

BlueSky: @plos.bsky.social

----------------------------------------------------

Comments from the reviewers (if applicable):

----------------------------------------------------

**Data Deposition**

If you have submitted a Research Article or Front Matter that has associated data that are not suitable for deposition in a subject-specific public repository (such as GenBank or ArrayExpress), one way to make that data available is to deposit it in the Dryad Digital Repository. As you may recall, we ask all authors to agree to make data available; this is one way to achieve that. A full list of recommended repositories can be found on our website.

The following link will take you to the Dryad record for your article, so you won't have to re‐enter its bibliographic information, and can upload your files directly:

http://datadryad.org/submit?journalID=pgenetics&manu=PGENETICS-D-26-00438R1

More information about depositing data in Dryad is available at http://www.datadryad.org/depositing. If you experience any difficulties in submitting your data, please contact help@datadryad.org for support.

Additionally, please be aware that our data availability policy requires that all numerical data underlying display items are included with the submission, and you will need to provide this before we can formally accept your manuscript, if not already present.

----------------------------------------------------

**Press Queries**

If you or your institution will be preparing press materials for this manuscript, or if you need to know your paper's publication date for media purposes, please inform the journal staff as soon as possible so that your submission can be scheduled accordingly. Your manuscript will remain under a strict press embargo until the publication date and time. This means an early version of your manuscript will not be published ahead of your final version. PLOS Genetics may also choose to issue a press release for your article. If there's anything the journal should know or you'd like more information, please get in touch via plosgenetics@plos.org.

# Editor report: Acceptance letter

**DOI:** 10.1371/journal.pgen.1012237.r004

**Authors:** Marnie E. Blewitt, Soni Lacefield

PGENETICS-D-26-00438R1

High-resolution global recombination mapping in C. elegans reveals sexual dimorphisms shaped by meiotic chromosomal features and structures

Dear Dr Libuda,

We are pleased to inform you that your manuscript entitled "High-resolution global recombination mapping in C. elegans reveals sexual dimorphisms shaped by meiotic chromosomal features and structures" has been formally accepted for publication in PLOS Genetics! Your manuscript is now with our production department and you will be notified of the publication date in due course.

The corresponding author will soon be receiving a typeset proof for review, to ensure errors have not been introduced during production. Please review the PDF proof of your manuscript carefully, as this is the last chance to correct any errors. Please note that major changes, or those which affect the scientific understanding of the work, will likely cause delays to the publication date of your manuscript.

Soon after your final files are uploaded, unless you have opted out or your manuscript is a front-matter piece, the early version of your manuscript will be published online. The date of the early version will be your article's publication date. The final article will be published to the same URL, and all versions of the paper will be accessible to readers.

For Research Articles, you will receive an invoice from PLOS for your publication fee after your manuscript has reached the completed accept phase. If you receive an email requesting payment before acceptance or for any other service, this may be a phishing scheme. Learn how to identify phishing emails and protect your accounts at https://explore.plos.org/phishing.

Thank you again for supporting PLOS Genetics and open-access publishing. We are looking forward to publishing your work!

With kind regards,

Sharmila Kamatchi

PLOS Genetics

On behalf of:

The PLOS Genetics Team

Carlyle House, Carlyle Road, Cambridge CB4 3DN | United Kingdom

plosgenetics@plos.org | +44 (0) 1223-442823

plosgenetics.org | Twitter: @PLOSGenetics
